# Environmental induction and phenotypic retention of adaptive maternal effects

**DOI:** 10.1186/1471-2148-8-3

**Published:** 2008-01-09

**Authors:** Alexander V Badyaev, Kevin P Oh

**Affiliations:** 1 Department of Ecology and Evolutionary Biology, University of Arizona, Tucson, AZ 85721, USA

## Abstract

**Background:**

The origin of complex adaptations is one of the most controversial questions in biology. Environmental induction of novel phenotypes, where phenotypic retention of adaptive developmental variation is enabled by organismal complexity and homeostasis, can be a starting point in the evolution of some adaptations, but empirical examples are rare. Comparisons of populations that differ in historical recurrence of environmental induction can offer insight into its evolutionary significance, and recent colonization of North America by the house finch (*Carpodacus mexicanus*) provides such an opportunity.

**Results:**

In both native (southern Arizona) and newly established (northern Montana, 18 generations) populations, breeding female finches exhibit the same complex adaptation – a sex-bias in ovulation sequence – in response to population-specific environmental stimulus of differing recurrence. We document that, in the new population, the adaptation is induced by a novel environment during females' first breeding and is subsequently retained across breeding attempts. In the native population, first-breeding females expressed a precise adaptive response to a recurrent environmental stimulus without environmental induction. We document strong selection on environmental cue recognition in both populations and find that rearrangement of the same proximate mechanism – clustering of oocytes that become males and females – can enable an adaptive response to distinct environmental stimuli.

**Conclusion:**

The results show that developmental plasticity induced by novel environmental conditions confers significant fitness advantages to both maternal and offspring generations and might play an important role not only in the successful establishment of this invasive species across the widest ecological range of extant birds, but also can link environmental induction and genetic inheritance in the evolution of novel adaptations.

## Background

Evolutionary biology is concerned with explaining the origin and diversification of organismal forms. However, despite great advances in the understanding of maintenance and adaptive evolution of existing organismal forms, we still know very little about their origin [1,2]. Especially puzzling is the origin of complex adaptations that involve close and context-dependent integration of multiple organismal systems.

The dual effect of a novel environment on phenotypic plasticity – simultaneous exposure of "hidden" developmental variation and strong selection on this variation – can be a starting point in the evolutionary persistence of some adaptations [3-6]. When individuals vary in their response to the novel stimulus, when this variability is heritable, and the stimulus is recurrent, such environmental induction can lead to eventual genetic determination of a novel adaptation [7,8]. The central thesis of this view is that evolutionary novelty often involves reorganization of preexisting phenotypes [9,10] and this results in similarity of the novel changes among individuals, facilitates response to novel selection pressures, and can ultimately lead to genetic assimilation of the novel trait [9,11-16]. Yet, empirical documentation of evolutionary persistence of environmentally induced adaptations is rare in natural populations [17-19].

**Figure 1:**
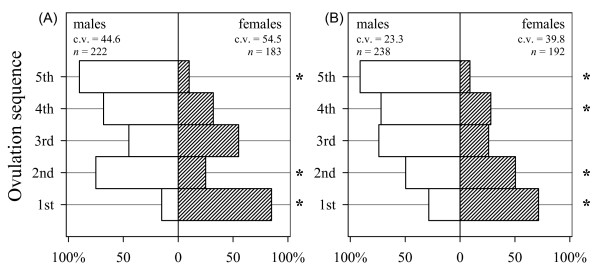
**Short-term sex-bias in ovulation sequence in house finch females**. Sex-bias in response to **A) **ambient temperature below egg-viability threshold during early breeding season in Montana (n = 86 nests), and **B) **late season nest mite infestation in Arizona (n = 110 nests). In both populations, there was no sex-bias in ovulation sequence in other parts of the breeding season. Asterisks show sex-ratios significantly deviating from parity. Coefficient of variation (cv) indicates variability in relative ovulation sequence of male and female eggs (see Methods).

Documentation of environmental induction and phenotypic retention of adaptive plasticity is the first step in investigating this proposed sequence, and rapid colonization of North America in the last 70 years by the house finch (*Carpodacus mexicanus*) – a species native to southwestern United States – provides such an opportunity. In both native (southern Arizona) and newly established (northern Montana) populations, sex-bias in ovulation sequence (Figure 1) confers significant fitness benefits – in the recently established population, it increases phenotypic variance in offspring growth which leads to greater juvenile survival under novel ecological conditions, and in the native population, it lessens offspring exposure to ectoparasites and associated mortality [20-22]. In Montana, the environmental stimulus to sex-biased ovulation is closely associated with the number of days during oogenesis when the ambient temperature falls below 4°C – minimum egg-tolerance temperature for most passerine birds ("critical temperature days" hereafter; [23]). In the native population in Arizona, the environmental cue is exposure of females during egg-laying to hematophagous ectoparasitic nest mite *Pellonyssus reedi *that infests most nests for 1.5–2 months during the late part of each breeding season [21]. The cues are unique for each population – Montana population is not exposed to nest mites, whereas Arizona population is not exposed to below egg-tolerance temperature during oogenesis and egg-laying.

Here we studied the similarity in responses to population-specific environmental stimulus and proximate mechanisms behind such responses in first-breeding (i.e., 8–12 months old) females in each population. Because most first-breeding females in the recently-established Montana population are long-distance immigrants from southern populations [24], they are not expected to have either an evolved recognition of the local environmental stimulus at the northernmost part of the species' range or an evolved and precise modification of ovulation sequence in response to this stimulus. On the other hand, first-breeding females in the Arizona population are mostly locally born or short-distance immigrants from local populations that experience yearly seasonal mite infestation that exerts high nestling mortality [21], and thus are expected to have evolved both a recognition of the local environmental stimulus (onset of mite season) and a response to it.

## Results

### Response to environmental stimulus

**Figure 2:**
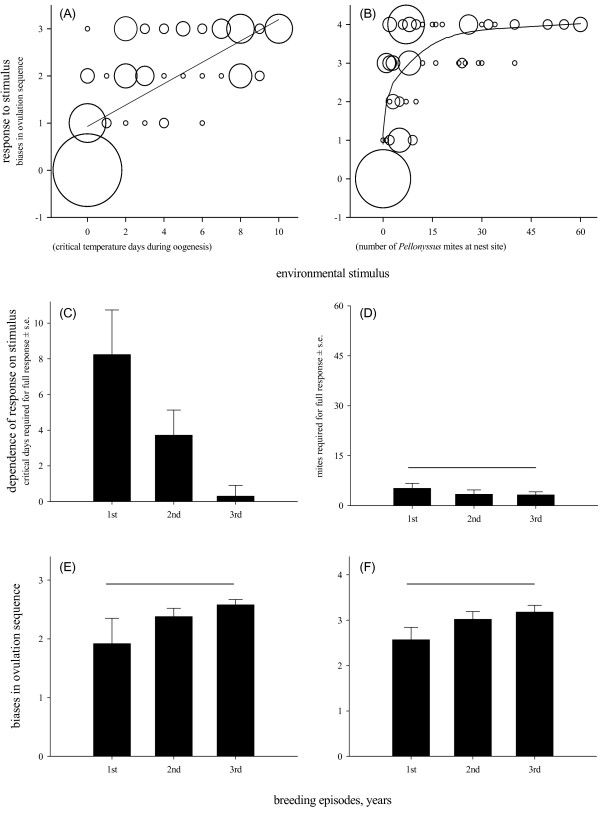
**The relationship between environmental stimulus (critical temperature days during oogenesis in Montana and number of mites at nest sites in Arizona) and response to the stimulus (number of biases in ovulation sequence) **in **A) **first-breeding females in Montana (n = 93 females), **B) **first-breeding females in Arizona (n = 131 females). **C) **Estimated number (mean ± s.e.m.) of critical temperature days during oogenesis required to exert full response (three biases in ovulation sequence) across female's lifetime in Montana (n = 51 females), **D) **Estimated number (mean ± s.e.m.) of nest mites during oogenesis required to exert full response (four biases in ovulation sequence) across female lifetime in Arizona (n = 29 females). Note that the ordinate axes in **C) **and **D) **are scaled identically to the abscissa axes in **A**) and **B) **to show the full range of the stimulus. **E) **Biases (mean deviations ± s.e.m.) in ovulation sequence across three breeding episodes of the same females in Montana, and **F) **in Arizona. Bubble radius is proportional to the number of overlapping data points. Lines connect means that are not significantly different.

Sex-bias in ovulation sequence of first-breeding females was closely associated with the number of critical temperature days during oogenesis in Montana (MT hereafter; maximum likelihood estimation χ^2 ^= 45.28, *p *< 0.001) and with number of mites at the nest site in Arizona (AZ hereafter; χ^2 ^= 106.3, *p *< 0.001), but the shape of the relationship differed between the populations. Linear regression best described the relationship between the response and stimulus for first-breeding females in MT (Figure 2a; biases = 0.93 ± 0.12 (s.e.m.) + 0.22 ± 0.02 critical days, t = 9.64, n = 93, *p *< 0.001; cubic spline fit *versus *linear regression fit: t = 4.66, *p *< 0.01), whereas a threshold-like cubic spline best described the relationship in first-breeding females in AZ (Figure 2b, χ^2 ^= 25.3, n = 131, *p *< 0.01). First-breeding females in MT had less precise sex-bias of ovulation sequence than first-breeding females in AZ (Figure 1; CV of within-sex variability of ovulation positions – males: 44.6% (MT) vs. 23.3% (AZ), females: 54.5 vs. 39.8%; both Fs > 3.38, *p *< 0.05).

Covariation between sex-bias and environmental stimulus across breeding episodes differed between the populations (Figure 2cd; interaction: population x breeding episode x relative stimulus, F_1,243 _= 5.98, *p *= 0.015). In MT, threshold for environmental induction of sex-bias decreased across breeding episodes in females that were followed throughout their lifetime (Figure 2c; χ^2 ^= 8.05, n = 51, *p *= 0.01), and by the third breeding episode a single critical temperature day during oogenesis was sufficient to exert a full response (Figure 2c). However, the strength of the response did not differ across breeding episodes across females' lifetime (Figure 2e; χ^2 ^= 4.33, *p *= 0.12). In AZ, neither the relationship between cue and response (Figure 2d; χ^2 ^= 0.84, n = 29, *p *= 0.51), nor the strength of the response differed across breeding episodes (Figure 2f; χ^2 ^= 3.54, *p *= 0.14).

### Natural selection on response to environmental stimulus

**Figure 3:**
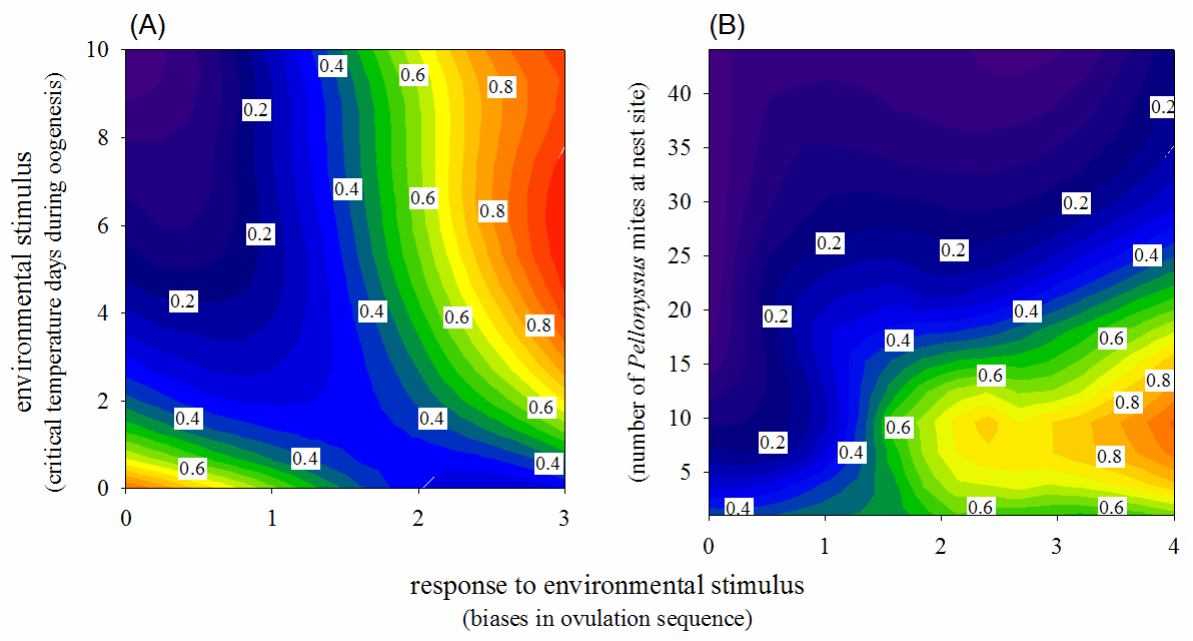
**Estimated contour plots of offspring survival as a function of number of deviations (biases) in ovulation sequence**. Response to **A) **critical temperature days in Montana population (n = 128 nests), and **B) **number of mites at nest site in Arizona population (n = 96 nests infested with mites). Note that the ordinate axis shows number of mites at the stage of egg-laying and this number increases greatly by the time nestlings hatch and mite-induced mortality occurs. Numbers show proportion of nestlings fledged out of the number of eggs laid.

We documented strong selection on response to environmental stimuli in both populations (Figure 3). In MT, first-breeding females that biased ovulation order when experiencing critical temperature days during oogenesis had the highest fledging success, whereas females that experienced the critical temperature days but did not bias ovulation sequence had the lowest success (Figure 3a; standardized selection differentials: b_ST _(bias) = 0.43, t = 5.25, *p *< 0.01; b_ST _(stimulus) = -0.13, t = -1.46, ns). In AZ, first-breeding females that responded strongly to the mites had the highest fledging success, whereas females that had lesser or no response had the lowest success (Figure 3b; b_ST _(bias) = 0.42, t = 6.21, *p *< 0.01, b_ST _(stimulus) = -0.39, t = -5.82, *p *< 0.01). The strength of selection on response to stimulus differed between the populations (interaction: population x relative stimulus x response: F_1,223 _= 56.15, *p *< 0.001, interaction: cue x population: F_1,223 _= 27.51, *p *< 0.001; model F_7,223 _= 25.01, *p *< 0.01), however the sharper peak of the estimated AZ fitness contour was likely confounded by the direct mortality effect of nest mites on nestling survival in nests with more than 20 mites at the onset of incubation (Figure 3b).

### Proximate mechanisms of response to environmental cue

**Figure 4:**
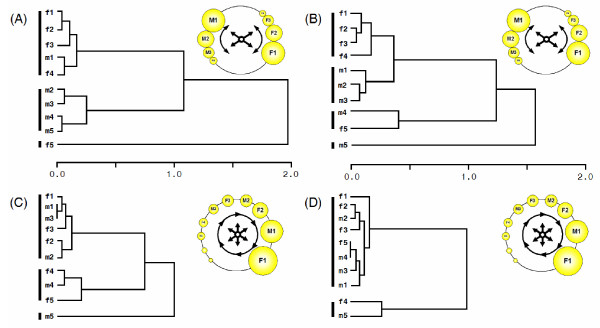
**Groups of oocytes similar in yolk (x-axis – Ward's minimum distance) in relation to oocyte' sex and ovulation order in first-breeding females**. **A) **Arizona population under mite infestation conditions (n = 72 nests), **B) **Montana population with > 5 critical days during oogenesis (n = 63 nests), **C) **Arizona population under mite free conditions (n = 99 nests), **D) **Montana population with ≤ 1 critical days during oogenesis (n = 34 nests). Drawings show hypothetical arrangement of oocytes in the ovary that would correspond to sex-specific clusters in **A) **and **B) **or non-sex specific hierarchical arrangement in **C) **and **D)**. Vertical bars on the left side delineate significantly distinct clusters.

To investigate the proximate mechanisms behind adaptive sex-biased ovulation, we studied similarity in yolk uptake among oocytes within and between sexes within a clutch in both populations. Specifically we examined whether oocytes that become males and females were recruited into the rapid growth stage at different times during oogenesis (see ***Background to avian oogenesis ***below; Figure 4a, c), a pattern expected to generate stronger correlations of yolk partitioning among single-sex follicles compared to mixed-sex follicles, or whether male and female oocytes are recruited in random order (Figure 4bd), a pattern not expected to generate sex-specific groups of oocytes similar in yolk deposition [see [25] for details]. In first-breeding females in AZ, our analyses revealed three significantly different, mostly sex-specific, oocyte groups in females that experienced mite infestation and had sex-biased ovulation order (Figure 4a; pseudo-t^2 ^= 10.01, *p *< 0.001), and three weakly differentiated, mixed-sex groups in females that breed under mite-free conditions and did not bias ovulation order (Figure 4b; pseudo t^2 ^= 5.76, *p *< 0.05; difference: Wilks' 8 = 0.91, F = 3.06, *p *< 0.05). In MT, there were four sex-specific groups in females that experienced critical temperature days during oogenesis (pseudo-t^2 ^= 29.5, *p *< 0.05), and two weakly differentiated, mixed-sex groups in females that did not experience critical temperature days during oogenesis (pseudo-t^2 ^= 8.3, *p *< 0.05; difference: Wilks' 8 = 1.16, F = 4.36, *p *< 0.05).

## Discussion

In order for environmentally-induced plasticity to have long-term evolutionary consequences, it has to be allowed and retained by organismal homeostatic processes, have a heritable component, induce similar changes across individuals, and confer consistent fitness benefits [6,9,26]; however, studies of these requirements are rare in natural systems. In several populations of house finches across North America, modification of within-clutch laying sequence of male and female eggs is under current natural selection in both maternal and offspring generations; these modifications are thought to be proximately produced by interplay of induced changes in maternal hormonal profile during breeding in novel or stressful conditions and associated distinct accumulation of hormones by oocytes that become males or females [23,27]. Here we showed that, first, the sex-biased ovulation sequence in first-breeding females in the newly established population was induced approximately proportionally to novel environmental stimulus, whereas first-breeding females in the native population responded to the environmental stimulus in a precise threshold-like pattern and without induction (Figure 2). Second, the dependence of response on environmental stimulus lessened across a females' lifetime in the newly established population (Figure 2c). Third, in both populations, sex-biased ovulation order was associated with the same mechanism – sex-specific groups of oocytes most likely induced through temporal similarity in recruitment time between oocytes that become the same sex (Figure 4; [25]).

These results raise two main questions. First, what are the mechanisms enabling phenotypic accommodation of environmentally-induced response across a female's lifetime? Second, how can environmental induction of ovulation sequence lead to evolution of precise patterns of sex-biased ovulation sequence found in some birds, including in the ancestral population of the study species? We also discuss alternative explanations for the observed findings, including the evolution of reaction norms in maternal traits between populations.

In both populations, the response to distinct environmental cues was produced by rearrangement of the same proximate mechanism – clustering of male and female oocytes. We suggest that such clustering is induced by temporal hormonal fluctuations in female's plasma, such that the effect of ambient temperature on prolactin-regulated female's incubation behaviors or direct experience of nest mites at the onset of mite infestation season, induces female hormonal fluctuations, resulting in exposure of growing oocytes to distinct hormonal profiles, subsequent accumulation of distinct hormonal concentrations which in turn, can affect the sex-determining meiotic division of oocytes [25,27-29]. Indeed, because hormonal mechanisms are involved in *both *assessment of environmental change and incorporation of novel environmental input, environmental induction of hormonal changes and subsequent phenotypic retention of their effects is a frequently documented route in the evolution of novel adaptations and morphologies [8,30-33]. Because of its complexity, redundancy, and environmental sensitivity, hormonal regulation of avian reproductive system is particularly well-suited for retention of environmentally-induced modifications [34]. Specifically, hormones regulating oocyte proliferation and ovulation have strong environmental sensitivity, and hormonally-mediated changes in gene expression as a result of prior breeding experience, changes in photoperiod, food, mate familiarity, or ambient temperature are often documented [35-39]. Moreover, environmental modifications of the first ovulation sequence, such as sensitivity to ovulation-inducing hormones, might be retained throughout a female's lifetime in vertebrates [40-42], apparently by the homeostatic effects of complex reproductive systems. Thus, hormonal regulation might have a major role in the observed rearranging the same mechanism for novel inputs and novel functions in the two study populations.

Female birds can show precise and context-dependent adjustment of sex-bias in laying order in relation to changes in mate quality, food availability, and time of breeding season, both across individuals [43-46] and between breeding attempts [47-49]. How can precise sex-bias in ovulation sequence evolve, especially when its initial induction is likely to be imprecise (e. g., Figures 1a, 2e)? We propose that evolved precision in sex-bias is caused by the linkage between hormonal mechanisms that influence sex-determination and mechanisms that enable distinct accumulation of hormones in oocytes that become males and females. Distinct allocation of hormones into male and female oocytes is under strong selection on offspring growth; for example in the two recently established house finch populations with opposite sex-bias in ovulation sequence, males produced in female-biased positions and females in male-biased positions accumulated hormones incompatible with their normal development [50], likely accounting for strong selection for environmental stimulus recognition documented in this study (Figure 3a). Because hormones in female plasma change with the progress of oogenesis, laying, and incubation, oocytes develop in and accumulate different hormonal milieus depending on the time of their sequestration, which, in turn, might produce temporal bias in time of sequestering of oocytes that become males and females resulting in sex-specific groups of oocytes described here [see also [51-53]]. Thus, natural selection should maintain close integration between the mechanisms by which hormones can bias sex-determination (including through environmental induction) and mechanisms enabling sex-specific accumulation of hormones by oocytes. Such integration not only can explain the observed within-generation increase in precision of sex-bias in ovulation sequence (Figure 2e), but also might be the mechanism for precise sex-specific maternal allocation of resources within a clutch in some birds [54-56].

In the native population, the finding of higher precision, greater expression, and lesser dependency of sex-bias in ovulation sequence on environmental induction (Figure 1, 2d) is consistent with observations that sex-bias in ovulation is accompanied by precise maternal adjustment of offspring growth, likely representing an evolved mother-offspring co-adaptation under selection on offspring morphology during periodic mite infestation [21]. On the contrary, in the new population, selection on modification of ovulation sequence acted primarily on the maternal generation, such that females that were able to resolve the contrasting hormonal requirements of overlapping incubation and oogenesis under novel breeding conditions had the highest fitness [23]. Elsewhere, we analyzed the effects of maternal adaptation on offspring across ten consecutive generations in relation to similarity in environmental conditions between maternal and offspring generations and found that the effects of induced maternal adaptation on the offspring induced wider phenotypic plasticity in offspring development and morphology rather than precise adjustment of offspring growth [22].

Alternatively, the newly established population might be expressing only a variant of the reaction norm that cannot be expressed in the native population due to more consistent and stronger selection on the reaction norm there [57-59]. In the context of this study system, the reaction norm is the sensitivity to environmental stimuli or a narrower range of adaptive response in the native population. However, strong within-individual changes in phenotypic plasticity (Figure 2c), the precise and complex nature of adaptive response where a different sex-bias in ovulation sequence is favored in different populations (Figure 1), and the fact that stimuli are unique for each population make it unlikely that the observed population differences in dependence of the response on the stimulus (Figure 2c, d) constitute differential expression of the same reaction norm or a retained response. At the same time, it is important for future studies to address the within-population contribution of genetic and environmental factors to the evolution of reaction norms.

## Conclusion

Across dispersing and native populations of the house finch, we observe differential importance of maternal effects for the evolution of local adaptations – from environmentally-induced maternal effects that increase developmental plasticity in the first few generations of the newly established population to the reliable production of locally adaptive morphologies in the absence of sex-biased maternal effects, but short-term and reversible maternal effects on offspring growth under mite infestation in the native population [20-22]. We showed that developmental plasticity induced by novel environmental conditions confers significant fitness advantages to both maternal and offspring generations and might play an important role not only in the successful establishment of house finches across the widest ecological range of extant bird species, but also can provide an example of the link between environmental induction and genetic inheritance in the evolution of novel adaptations.

## Methods

### Study populations and general methods

House finches were studied in 1995–2006 in the recently established population at the northernmost part of species' range in northwestern Montana, where this species started breeding in the late 1970s, and in 2002–2007 in the southern part of their native range in southwestern Arizona, 2700 km to the south, where finches bred for at least 10,000 years. In both populations, all resident birds were marked with a unique combination of four rings, and age category and prior breeding experience were known for all birds included in this study. All females laid one egg per day between 0500 and 1100 and eggs were numbered sequentially on the day of laying. Embryos or nestlings were sexed molecularly [60] and the maximum oogenesis duration (ten days concluding with the laying of penultimate egg and ovulation of the last egg) was assessed with the oocyte lipid accumulation method [61]. To minimize the effect of clutch size on sex-bias in ovulation sequence, we restricted the analyses to 4 and 5 egg clutches. Analysis of similarity of oocyte yolk uptake was conducted according to [[25], see also below]. To examine changes in response across breeding attempts we followed the same females across their lifetime.

### Environmental stimulus and response measures

In the MT population, we recorded the number of days during a ten-day oogenesis period with average daily (24 hr) temperature ≤ 4°C ("critical temperature days"). To minimize the effect of timing of this environmental stimulus on probability of response, we excluded four nests where the stimulus was absent during the first five days of oogenesis. Weather data were obtained from permanent weather stations at the Missoula Country Airport one km from the MT study site [28]. In Arizona population, we counted *Pellonyssus reedi *mites at nest sites every second day and the total population of mites was subsequently verified by fumigating nests with chloroform; only estimated abundance of nest mites at the egg-laying stage was used in this study. Because the full adaptive response – population-specific ovulation sequences of male and female eggs – was known for each population ([20,21], which see for tests of sex-biased ovulation), we measured the magnitude of the response as the number of "correct" deviations in each ovulation sequence (i.e., clutch) following [23]. Briefly, full "correct" response was three positions deviated from parity (1^st^, 2^nd^, and the last) for the MT population and four deviated positions (1^st^, 2^nd^, 4^th ^and last) for the AZ population (Figure 1). To compare populations, we divided the number of deviations ("biases" hereafter) for each female by the maximum number of deviations for the population. All analyses involving this measure were conducted with nest identity as a random effect to correct for female-specific ovulation sequences. Variability in the precision of sex bias in ovulation sequences (Figure 1) was measured by ranking male and female egg-laying positions separately within a clutch and calculating coefficient of variation for mean within-clutch probability position for each sex [see also [62]] that was subsequently compared with Levene's test.

### Brief background to avian oogenesis

Ovarian oocytes recruited into the pre-ovulatory pool undergo rapid yolk accumulation and typically form temporal hierarchy of growth, followed by sequential ovulation. In addition to temporal variation in recruitment to the rapid yolk deposition stage, hierarchical arrangements among the simultaneously growing oocytes and ovulation intervals are maintained by growth inhibiting hormonal interactions among maturing oocytes [63-65]. Such inhibiting interactions have pronounced spatial patterns, such that only follicles in the close proximity or at similar stages of development are affected. Thus, differences between oocytes that become males and females in either time of recruitment or in spatial arrangement in the ovary can produce sex-specific groups or "clusters" of oocytes. Such clusters have been inferred through analyses of similarity in oocyte accumulation of lipids, carotenoids, vitamins, and hormones [25,27,50].

### Statistical analyses

To assess shape and magnitude of the response (e.g., bias number) as a function of environmental stimulus (number of critical days in MT and number of mites at nest site in AZ) we used regression procedures in generalized additive models of PROC GAM in SAS 9.13. The Poisson regression analysis of GAM procedure enables simultaneous test of the stimulus, estimation and statistical comparison of the best shape of the relationship between the stimulus and the response, and visual assessment of the value of stimulus corresponding with the full response. To estimate and test the change in dependency of the response on stimulus, we plotted, with ODS graphics module of PROC GAM, for each breeding episode in both populations, the 99% confidence interval around the best-fit curve. We then recorded the smallest value of the stimulus corresponding to the full response (three positions in MT and four in AZ). We repeated this procedure with replacement for all nests in MT and AZ datasets and mean ± s.e.m. were calculated for Figure 2c, d. Overall significance of the dependency of response on stimulus was tested with PROC GENMOD logistic regression, the means of response dependence on stimulus among breeding episodes were compared with Waller-Duncan K-ratio *t*-test, and the magnitude of response across breeding episodes was compared with repeated measures ANOVA in REPEATED module of PROC MIXED of SAS 9.13 with breeding episode, female age cohort, and year as categorical fixed effects and female identity as a random effect. To compare response to environmental stimulus across all females, breeding episodes, and populations in the single test, we standardized both the response and strength of stimulus variables to percentages of the full response and maximum stimulus (three biased positions, and 10 critical temperature days in MT and four biased positions and 55 mites in AZ) and tested the interaction between the factors with PROC GLM with the response constrained by female identity. Correlational structure of oocyte similarity in yolk uptake was converted to distances in canonical discriminant analysis (PROC CANDISC in SAS 9.13). The cluster analysis of similarity in correlational structure between follicles of different ovulation order and sex was conducted by Ward's minimum distance method using pseudo-*F *and preudo-*t*^2 ^statistics to estimate the number of statistically distinct clusters [after [25]].

## Authors' contributions

AVB designed the study and wrote the manuscript. AVB and KPO conducted field work and analyzed the data. Both authors read and approved the manuscript.
